# Transactions of the Scandinavian Society of Naturalists and Physicians, 1839

**Published:** 1840-10

**Authors:** 


ART. V.—*Forhandlingar vid det af Skandinaviska Naturforskare och Läkare Hällna möte i Gotheborg.* Aer 1839.—*Gotheborg*, 1840. 8vo, pp. 188.

*Transactions of the Scandinavian Society of Naturalists and Physicians held at Gottenburg, in the year* 1839.—*Gottenburg*, 1840.

THIS little volume, written in the Swedish language, gives an account of the meeting of savans at Gottenburg in July, 1839. It was attended by ninety-two members, who resolved themselves into sections of physics, natural history, and medicine, the latter of which consisted of forty-eight members, of whom Professor Holst, of Christiania, was president. Although this was the most considerable section of the meeting, little was done in it; indeed the whole seems to have been more distinguished for kindly feelings and good fellowship, than for severer and scientific proceedings.

Professor Eschricht, of Copenhagen, read a paper upon the origin of Entozoa. He was opposed to the opinion that these are primarily generated, not from ova at all, properly so called, but spontaneously in each organ of the animals in which they are found. He supported his views chiefly by the fact of each entozoon possessing proper organs of generation.

Dr. Sommer, of Copenhagen, read a paper upon the classification of diseases of the skin, in which he proposed to rearrange them into twelve natural groups, viz.:

1, Dermatoses exanthematicæ; 2, D. erysipelaceæ; 3, D. eczematosæ; 4, D. impetiginosæ; 5, D. scabiosæ; 6, D. papuliformes; 7, D. squamosæ;

8, D. folliculosæ; 9, D. favosæ; 10, D. cancrosæ et cancroideæ; 11, D. leprosæ; 12, D. syphiliticæ.

Dr. Cederschjold, of Stockholm, read a paper upon the position of the head of the child in utero; and Dr. Egeberg, of Christiania, related some cases in which he had performed the Stromeyerian operation. These are the chief subjects relating to medicine which were discussed —that by Dr. Sommer at considerable length.

There was a feature in this meeting quite novel and hitherto unheard of at similar reunions; and Heaven forefend that it should be otherwise in future. A Latin poem was recited! It defies translation; and it would almost seem as if each Professor, Lector, and Doctor, had furnished a line:—who was the Laureat does not appear. In the first stanza the dangers of the sea cannot separate the sons of science. In the next, the assembled conclave are the princes of the republic of letters, as Rome had once a senate of kings. In the third, they will keep the eternal fire alive; and the world, enlightened by them, will never be in darkness. In the fourth, they unite discordant nations; and the language of each is no longer foreign to their ears. And lastly, but not least, Gottenburg is the first city to witness the phenomenon; and the first also to pray to God to prosper the attempt.

